# Mapping intellectual structure and research hotspots of cancer studies in primary health care: A machine-learning-based analysis

**DOI:** 10.1097/MD.0000000000041749

**Published:** 2025-03-21

**Authors:** Muhammet Damar, Hale Turhan Damar, Şeyda Özbiçakci, Gökben Yasli, Fatih Safa Erenay, Güzin Özdağoğlu, Andrew David Pinto

**Affiliations:** a Dokuz Eylul University, İzmir, Türkiye; b Upstream Lab, MAP, Li Ka Shing Knowledge Institute, Unity Health Toronto, Toronto, Ontario, Canada; c Elderly Care Program, Health Services Vocational School, Izmir Democracy University, Izmir, Turkey; d Department of Public Health, İzmir Health Directorate, İzmir, Türkiye; e Management Science and Engineering, University of Waterloo, Waterloo, Ontario, Canada; f Department of Family and Community Medicine, Faculty of Medicine, University of Toronto, Toronto, Ontario, Canada.

**Keywords:** cancer, family medicine, intellectual structure, oncology, primary care, topic modeling

## Abstract

In the contemporary fight against cancer, primary health care (PHC) services hold a significant and critical position within the healthcare system. This study, as one of the most detailed investigations into cancer research in primary care, comprehensively evaluates cancer studies from the perspective of PHC using bibliometric techniques and machine learning. The dataset for the analyses was sourced from the Web of Science (WoS) Core Collection database on March 20, 2024. The Bibliometrix package within the R programming environment, alongside the Biblioshiny application, and VOSViewer software were employed for the bibliometric analyses. In this study, Latent Dirichlet Allocation was utilized as a prominent topic modeling algorithm. The implementation of this technique utilized Python along with the SciKit-Learn and Gensim libraries, ensuring robust model development and evaluation. The 2040 articles were produced by a total of 6705 different authors, 2166 different affiliations, and 75 different countries. Cancer survivors are more vulnerable and need more sensitive health services. The most intensively studied 3 cancer types in the PHC, listed by prevalence, are colorectal cancer, breast cancer, and cervical cancer. Additionally, prominent research topics in PHC include cancer screening, diagnosis, early detection, prevention, education, genetic factors and family history, risk factors, symptoms/signs, preventive medicine, referral and consultation, chronic disease management and health services research for cancer patients, health care disparities, palliative care, and communication with patients in PHC. Family physicians, being the first point of contact with the public, play a crucial role in preventing cancer cases, caring for patients with active cancer diagnoses, supporting cancer survivors in their post-cancer lives, and identifying and referring cancer cases at the earliest stages. However, cancer has many types, each with its own distinct symptoms, as well as similar types to each other. At this point, periodic educational training for doctors on cancer by health authorities, regular publication of cancer-related guidance resources by the central healthcare system, development of integrated decision support tools used by physicians during patient care, and the creation of informative mobile applications for cancer prevention or post-cancer life for patients have been considered highly critical.

## 1. Introduction

The importance of primary care within the overall healthcare system is well-recognized.^[1–4]^ One of the main duties of primary healthcare services lies in safeguarding public health by implementing diagnostic, preventive, and therapeutic measures against cancer, the second leading cause of death globally, and following up with patients after these measures.^[5–8]^ Family physicians and general practitioners, due to their frequent frontline interactions with the public, are ideally positioned to evaluate an individual’s distinct risk factors (e.g., hereditary and genetic factors, personal history, diet, etc), identify and refer to suitable diagnostic/preventive interventions, provide counseling, and discuss/plan health-promoting strategies tailored to patients’ risk factors. Thus, primary care providers significantly contribute to cancer prevention and detection from its earliest stages, the management and care of patients with active cancer diagnoses including continuous support and comprehensive care throughout the treatment process, and the follow-up at the post-treatment stage against recurrences and secondary cancers.^[6,9–12]^ These primary care services improve health outcomes for patients under risk and cancer survivors and reduce the burden of cancer.^[13]^

The main expectation from primary care is rapidly recognizing cases with cancer risk from symptoms and relevant risk factors, forwarding them to the appropriate interventions, and following them up after the tertiary or secondary care.^[14,15]^ A typical family physician encounters 3 to 4 new cancer diagnoses annually among their patients.^[16]^ Among all cases, 9 out of every 10 cancer patients present with symptoms, and the vast majority of these individuals initially seek consultation within the PHC system.^[11]^ Individuals diagnosed with cancer tend to visit their family physicians nearly twice as frequently compared to other patients, and it has been observed that they primarily consult their family physicians first rather than directly visiting oncology centers when their symptoms worsen.^[17,18]^

Although these figures underscore the significance of primary care for cancer management, there are challenges hindering the delivery of primary health services towards better cancer care. Various research activities have evolved around these issues; however, to the best of our knowledge, a comprehensive literature review evaluating the global impact of primary healthcare research on cancer is missing. This study aims to present a comprehensive assessment of cancer research in primary healthcare literature by employing machine learning and bibliometric methods to analyze articles published in high-quality journals within the field. Bibliometrics is a well-known methodology for analyzing the state of and scientific productivity within research fields.^[19–21]^ Its applications in public health and medicine have been growing including those in the field of PHC.^[22–24]^

## 2. Materials and methods

### 2.1. Data retrieval and research methodology

**Figure 1.**
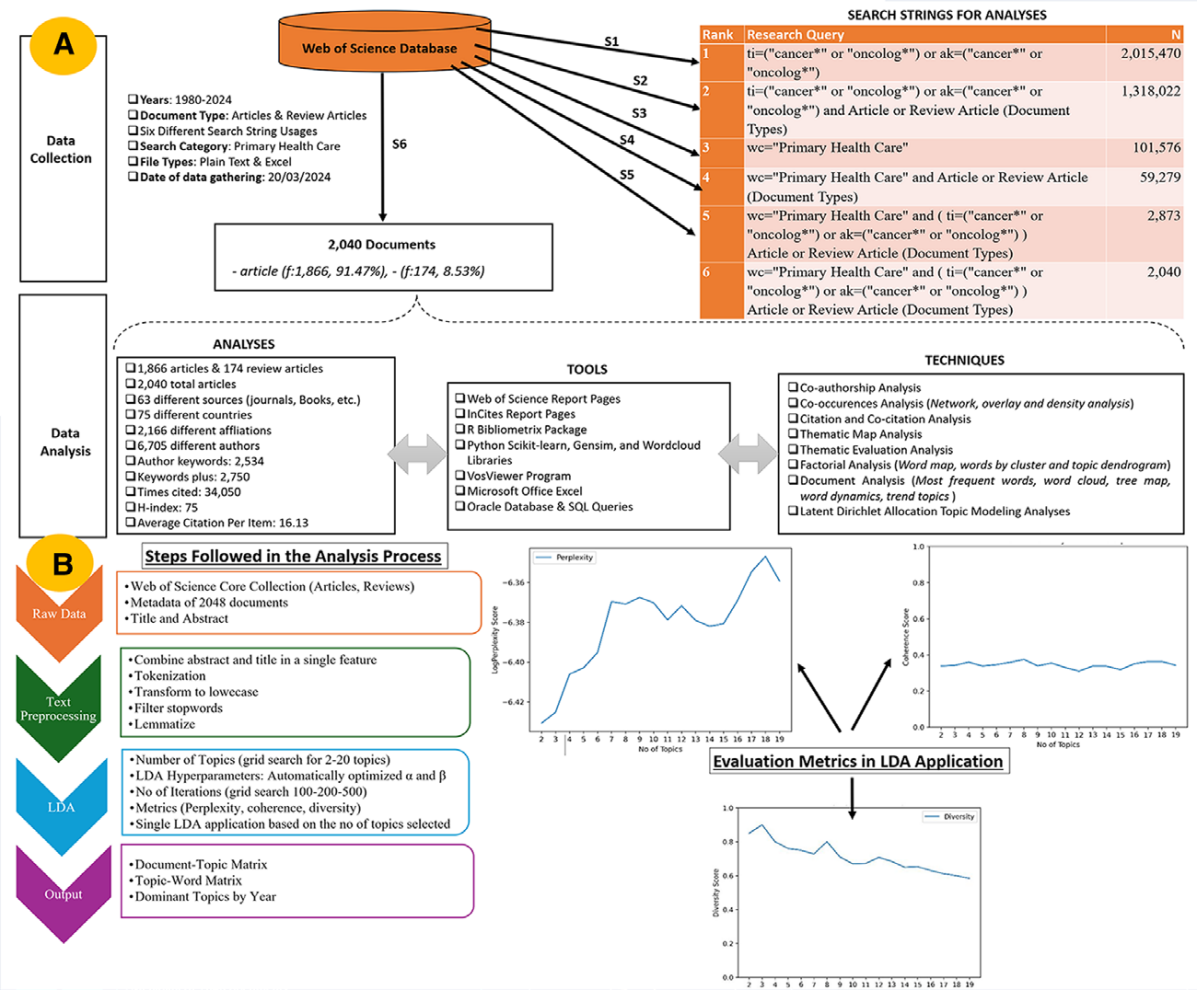
Search string and research methodology.

The dataset for the analyses was sourced from the Web of Science (WoS) Core Collection database on March 20, 2024. In this study, it was not necessary to obtain ethical committee approval for the use of data. The data utilized are publicly available and can be accessed through the WoS. The Bibliometrix package within the R programming environment, alongside the Biblioshiny application, and VOSViewer software were employed for the bibliometric analyses. Furthermore, data preprocessing tasks such as cleaning, filtering, joining, and aggregation were performed using spreadsheet tools and SQL operations, essential for the initial stages of such data science projects. Beside the convensional bibliometric analyses, a topic modeling approach was employed to extract more detailed insights from the content of articles published in the selected field. This involved the application of machine learning and text analytics techniques, specifically leveraging Latent Dirichlet Allocation (LDA). The comprehensive end-to-end methodology employed in this study, encompassing the text strings utilized during the data collection phase, the analyses and techniques conducted, and the network-based visualization tools selected, and preliminary meta-data is systematically depicted in Figure 1 as a detailed workflow.

Thomson Reuters WoS first introduced the “*Primary Health Care*” (PHC) subject category in 2011, indexing 13 journals in that year. This subject category enhances the understanding of scientific output in primary healthcare services and family medicine.^[25,26]^ Currently, WoS provides access to approximately 34,000 leading journals across various fields, supporting a broad spectrum of use cases to offering analytical datasets and granting access to raw data.^[27]^ This publication and data portfolio of WoS allows for the comprehensive mapping of research within the primary healthcare services in the cancer domain, leveraging modern and advanced analytical environments with the aid of machine learning and network models, and is the reason for its selection in this study.

### 2.2. Latent dirichlet allocation for topic modeling

Multiple techniques exist for deriving insights from text documents; in this study, we utilize LDA, a prominent topic modeling algorithm. LDA operates as a Bayesian network, positing that documents are composed of words that contribute to the determination of underlying topics. Consequently, each word in the document is probabilistically assigned to different topics, allowing for the mapping of documents to a coherent list of topics. Within the framework of text modeling, topic probabilities offer an explicit representation of the document’s thematic structure.^[26,28–30]^ LDA assumes that each document is a mixture of topics, with each topic characterized by a distribution over words. It is an effective tool for discovering thematic content. LDA can be applied in situations where the dataset is large and unstructured, particularly when the topics are not predefined and need to be uncovered through the data itself.^[21,26,29]^ The topic modeling methodology, particularly the LDA approach, encompasses 4 critical stages: data collection and preparation, LDA model construction, model evaluation, and result interpretation. Alternative methods to LDA are Non-negative Matrix Factorization (NMF),^[31]^ Latent Semantic Analysis (LSA),^[32]^ Hierarchical Dirichlet Process,^[33]^ and BERTopic.^[34]^ Each of these alternatives has its strengths and weaknesses, and the choice of method often depends on the specific nature of the dataset and the research objectives. It is particularly useful in text-based research such as academic articles, social media posts, customer reviews, and news articles. Additionally, our dataset appears to be of a suitable size for LDA analysis.

The abstracts and titles of 2048 articles are categorized using LDA to identify key topics and gain insights into the literature’s overall trajectory. The implementation of this technique utilizes Python along with the Spacy and Gensim libraries,^[26,35]^ ensuring robust model development and evaluation. This comprehensive approach enabled a detailed exploration and understanding of the thematic structures within the targeted research corpus. Figure 1b summarizes all stages applied from the raw dataset to the LDA phase.

LDA assumes each document is a mixture of topics, with words assigned to topics based on statistical properties of the Dirichlet distribution. It requires a dictionary and a corpus as inputs, where text data is tokenized, preprocessed, and indexed. LDA generates a Document-Topic Matrix and a Topic-Word Matrix, estimating word-topic probabilities using Gibbs Sampling for computational efficiency. The model’s performance is fine-tuned using metrics such as perplexity and coherence while hyperparameters and the number of topics are optimized accordingly. LDA produces 2 key outputs: The Topic-Word Matrix and the Document-Topic Matrix. The Topic-Word Matrix identifies *k* topics, each represented by the *n* most relevant words, facilitating topic interpretation. The Document-Topic Matrix, in contrast, captures the distribution of topics across documents, assigning probability values that indicate each document’s affiliation with specific topics. This is essential for determining the dominant topic of a document. These matrices significantly aid researchers in analyzing large-scale textual datasets. The Topic-Word Matrix supports topic labeling and enables a rapid assessment of overarching themes within the dataset. Meanwhile, the Document-Topic Matrix assigns probability values across topics for each document, allowing for systematic categorization and ranking based on topic relevance.^[29,30]^

A grid search is conducted to optimize hyperparameters, ensuring the model operates with the best parameter set. Perplexity, diversity, and coherence metrics^[36,37]^ are used to assess model quality, with recommended thresholds varying based on the research domain and context. Based on the analysis, the evaluation of perplexity, diversity, and coherence metrics (Fig. 1B) for optimized LDA hyperparameters across a range of 2 to 20 topics indicates that the data is best structured into 8 distinct topics.

The selection of the topic count is based on the point where the log-perplexity value first begins to decline, while coherence and diversity values remain relatively high. Due to the proximity of metric values, alternative topic counts may also be considered. As the number of topics decreases, different themes tend to merge, leading to overlapping subject areas. Conversely, while increasing the number of topics may improve certain metrics, it can also reduce within-group diversity and weaken the ability to form well-defined topic clusters.

Beyond LDA, various topic modeling methods are available.^[38,39]^ Several key factors influenced the selection of LDA as the preferred method. First, LDA allows for the predefined specification of the number of topics, enabling a more controlled and structured analysis. Additionally, its probabilistic framework, which acknowledges that documents can belong to multiple topics, aligns well with the objectives of this research.^[40]^ Alternative methods revealed certain limitations such as uncertainty in determining the optimal number of topics, or reliance on a linear decomposition mechanism reduced interpretability. LDA on different text representations such as word embeddings have also been evolved to handle relatively large and more complex text datasets, thus these models require intensive training data.^[41]^

## 3. Results

### 3.1. General statistics

Between 1980 and 2024, a total of 2040 articles (articles:1866, reviews:174) were produced in the field of PHC research on cancer. The year 2022 witnessed the highest number of publications with 132 articles. The h-index of these publications stands at 73. The studies were supported by 860 different funding organizations, with the top 4 being: United States Department of Health and Human Services (*N:* 137, 6.71%), National Institutes of Health United States (USA) (*N:* 163, 7.99%), National Cancer Institute - National Institutes of Health (*N:* 81, 3.97%), and Cancer Research United Kingdom (UK) (*N:* 75, 3.67%).

### 3.2. Authors, institutions, and country analyses

The 2040 articles were produced by a total of 6705 different authors, 2166 different affiliations, and 75 different countries. The international co-authorship rate stands at 9.41%, with an average of 4.48 coauthors per document and an average document age of 13.10 years. The top 5 most prolific researchers in the field of PHC cancer research are as follows: Willie Hamilton (University of Exeter Medical School, Average Citation Per Article (*ACPA*): 26.52, *N:* 64), Peter Vedsted (Aarhus University, *ACPA*: 5.78, *N:* 36), Jon D. Emery (University of Melbourne, *ACPA*: 21.52, *N:* 33), Fiona Walter (University of Cambridge, *ACPA*: 23.09, *N:* 32), and Richard Neal (University of Exeter, *ACPA*: 31.71, *N:* 24) (Appendix S1, Supplemental Digital Content, http://links.lww.com/MD/O556). When analyzing the articles by institution, the top 5 institutions are: University of Exeter (England, *N:* 81, 3.97%), University of London (England, *N:* 79, 3.87%), University of Toronto (Canada, *N:* 75, 3.67%), University of California System (USA, *N:* 59, 2.89%), and University of Oxford (England, *N:* 54, 2.64%) (Appendix S2, Supplemental Digital Content, http://links.lww.com/MD/O558). Over the years, the top 5 most productive countries have been identified as follows: USA (*N:* 716, 35.09%), England (*N:* 318, 15.58%), Canada (*N:* 224, 10.98%), Australia (*N:* 166, 8.13%), and Poland (*N:* 88, 4.31%) (Appendix S3, Supplemental Digital Content, http://links.lww.com/MD/O559). Additionally, the collaborations, clusters, and time-based changes among researchers (Appendix S1, Supplemental Digital Content, http://links.lww.com/MD/O556), institutions (Appendix S2, Supplemental Digital Content, http://links.lww.com/MD/O558), and countries (Appendix S3, Supplemental Digital Content, http://links.lww.com/MD/O559) can be observed in the provided supplementary documents.

### 3.3. Document and journal analyses

The analyses revealed that 6705 unique authors published articles on this subject across 63 different journals, leveraging 10,042 distinct sources and citing 46,121 references. The average number of citations per document stands at 16.13. Remarkably, 53.62% of these articles (*N:* 1094) were made available as open access. Table 1 illustrates the journals with the highest frequency of cancer research publications in the PHC domain, while Table 2 highlights the most cited articles in this field. Furthermore, according to Bradford’s Law,^[42]^ the primary journals in PHC research that are essential for tracking cancer-related studies include the British Journal of General Practice, Family Practice, Canadian Family Physician, and American Family Physician.

**Table 1:** Top twenty-five journals for high-volume cancer research publications in PHC research field

Rank	Journal	5JIF	Research domain	SCIE/ESCI	ACPA	C	N	%
1	British Journal of General Practice	6.40	Medicine, General & Internal; Primary Health Care	SCIE	25.36	6390	252	12.35
2	Family Practice	2.60	Medicine, General & Internal; Primary Health Care	SCIE	20.62	3711	180	8.82
3	Canadian Family Physician	3.90	Medicine, General & Internal; Primary Health Care	SCIE	13.73	2457	179	8.77
4	American Family Physician	7.40	Medicine, General & Internal; Primary Health Care	SCIE	29.92	5116	171	8.38
5	Primary Care	3.60	Medicine, General & Internal; Primary Health Care	SCIE	10.72	1276	119	5.83
6	Journal of The American Board of Family Medicine	2.90	Medicine, General & Internal; Primary Health Care	SCIE	17.77	2026	114	5.58
7	Journal of Family Medicine and Primary Care	–	Primary Health Care	ESCI	3.13	341	109	5.34
8	BMC Family Practice	3.30	Medicine, General & Internal; Primary Health Care	SCIE	17.37	1720	99	4.85
9	Family Medicine and Primary Care Review	0.50	Primary Health Care	ESCI	0.64	57	89	4.36
10	Journal of Family Practice	0.60	Medicine, General & Internal; Primary Health Care	SCIE	26.79	2063	77	3.77
11	Annals of Family Medicine	5.50	Medicine, General & Internal; Primary Health Care	SCIE	44.07	3173	72	3.52
12	Scandinavian Journal of Primary Health Care	2.50	Health Care Sciences & Services; Medicine, General & Internal; Primary Health Care	SCIE	11.21	762	68	3.33
13	Australian Family Physician	1.177	Medicine, General & Internal; Primary Health Care	SCIE	8.68	547	63	3.08
14	Korean Journal of Family Medicine	2.20	Primary Health Care	ESCI	7.55	453	60	2.94
15	Journal of Primary Care and Community Health	3.50	Primary Health Care	ESCI	6.48	350	54	2.64
16	Australian Journal of General Practice	2.10	Medicine, General & Internal	SCIE	8.39	302	36	1.76
17	Australian Journal of Primary Health	1.60	Health Care Sciences & Services; Health Policy & Services; Primary Health Care; Public, Environmental & Occupational Health	SCIE	6.57	197	30	1.47
18	BMC Primary Care	–	Medicine, General & Internal; Primary Health Care	SCIE	1.07	29	27	1.32
19	Family Medicine and Community Health	4.30	Primary Health Care	ESCI	3.61	83	23	1.12
20	Journal of Primary Health Care	1.30	Primary Health Care	ESCI	6.57	151	23	1.12
21	Atencion Primaria	1.90	Medicine, General & Internal; Primary Health Care	SCIE	3.32	73	22	1.07
22	European Journal of General Practice	6.50	Medicine, General & Internal; Primary Health Care	SCIE	6.90	138	20	0.98
23	Family Medicine	2.00	Medicine, General & Internal; Primary Health Care	SCIE	39.50	790	20	0.98
24	Primary Health Care Research and Development	2.10	Primary Health Care	SCIE	7.32	139	19	0.93
25	BJGP Open	–	Primary Health Care	ESCI	2.18	37	17	0.83

5JIF = Five Year Journal Impact Factor, ACPA = Average Citation per Articles, C = Citation Count, N = Article Count.

**Table 2:** Top twenty most cited cancer research articles in PHC research field

Rank	Title	Journal	5JIF	Authors	Year	C
1	Lung cancer: Diagnosis and management	American Family Physician	7.4	Collins, LG; Haines, C; (...); Enck, RE	2007	373
2	Targeted therapies: A new generation of cancer treatments	American Family Physician	7.4	Gerber, DE	2008	304
3	Health care of young adult survivors of childhood cancer: A report from the childhood cancer survivor study	Annals of Family Medicine	5.5	Oeffinger, KC; Mertens, AC; (...); Robison, LL	2004	294
4	Treatment of Breast Cancer	American Family Physician	7.4	Maughan, KL; Lutterbie, MA and Ham, PS	2010	268
5	Early detection and treatment of skin cancer	American Family Physician	7.4	Jerant, AF; Johnson, JT; (...); Caffrey, TJ	2000	264
6	Diagnosis and Management of Endometrial Cancer	American Family Physician	7.4	Braun, MM; Overbeek-Wager, EA and Grumbo, RJ	2016	246
7	Esophageal Cancer	American Family Physician	7.4	Short, MW; Burgers, KG and Fry, VT	2017	237
8	Long-Term Psychosocial Consequences of False-Positive Screening Mammography	Annals of Family Medicine	5.5	Brodersen, J and Siersma, VD	2013	236
9	Diagnosis and Management of Ovarian Cancer	American Family Physician	7.4	Doubeni, CA; Doubeni, ARB and Myers, AE	2016	208
10	Lay understanding of familial risk of common chronic diseases: A systematic review and synthesis of qualitative research	Annals of Family Medicine	5.5	Walter, FM; Emery, J; (...); Marteau, TM	2004	200
11	Comparison of breast cancer patient satisfaction with follow-up in primary care versus specialist care: results from a randomized controlled trial	British Journal of General Practice	6.4	Grunfeld, E; Fitzpatrick, R; (...); Vessey, M	1999	193
12	Ovarian Cancer: An Overview	American Family Physician	7.4	Roett, MA and Evans, P	2009	185
13	Bladder Cancer: Diagnosis and Treatment	American Family Physician	7.4	DeGeorge, KC; Holt, HR and Hodges, SC	2017	183
14	Sedentary Lifestyle: Overview of Updated Evidence of Potential Health Risks	Korean Journal of Family Medicine	2.2	Park, JH; Moon, JH; (...); Oh, YH	2020	175
15	Pancreatic cancer: Diagnosis and management	American Family Physician	7.4	Freelove, R and Walling, AD	2006	172
16	Disclosing complementary and alternative medicine use in the medical encounter - A qualitative study in women with breast cancer	Journal of Family Practice	0.6	Adler, SR and Fosket, JR	1999	165
17	Screening for cervical cancer: A review of women’s attitudes, knowledge, and behavior	British Journal of General Practice	6.4	Fylan, F	1998	164
18	Breast and cervical cancer screening:: Impact of health insurance status, ethnicity and nativity of Latinas	Annals of Family Medicine	5.5	Rodríguez, MA; Ward, LM and Pérez-Stable, EJ	2005	15
19	Symptom burden among cancer survivors: Impact of age and Comorbidity	Journal of The American Board of Family Medicine	2.9	Mao, JJ; Armstrong, K; (...); Farrar, JT	2007	155
20	Bone Cancer: Diagnosis and Treatment Principles	American Family Physician	7.4	Ferguson, JL and Turner, SP	2018	150

5JIF = Five Year Journal Impact Factor, C = Citation Count.

### 3.4. Research domains and content analysis

**Figure 2.**
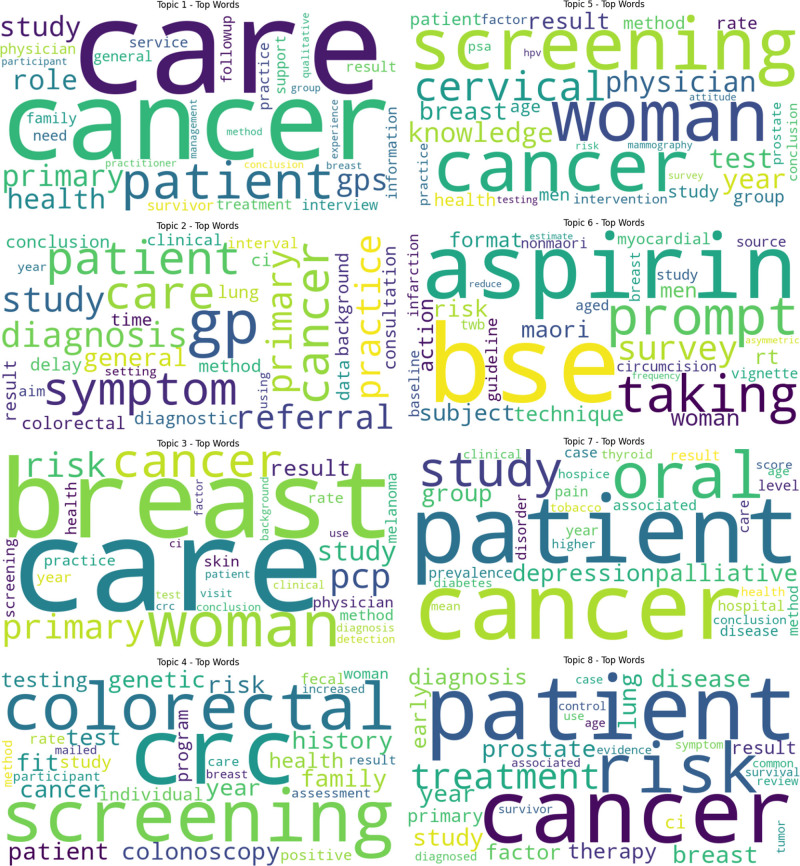
LDA topics summary (Abstracts and Titles).

Cancer-related studies within the PHC are also strongly associated with 5 other key research fields: Medicine general and internal (*N:* 1498), health care sciences and services (*N:* 101), public environmental and occupational health (*N:* 38), health policy and services (*N:* 33), and oncology (*N:* 26). To effectively summarize the cancer-related articles in the PHC literature and to identify underlying themes, a LDA topic model was employed (Fig. 2). The model, through analysis of textual data from publications, identified specific topics and themes under which meaningful data groups and insights have been gathered. These are depicted in Figure 2 with 8 distinct word clouds.

A detailed analysis and interpretation of the LDA topic analysis in Figure 2 leads to the derivation of the following 8 distinct topic groups:

- Patient consultations in primary care for cancer, the role of general practitioners, physicians, and family doctors in patient care, and the care process.
- Diagnosis of cancer in primary care, delays in diagnosis, referral status, and specific focus on lung and colorectal cancer.
- Common cancers in women include breast cancer and skin cancer.
- The importance of family history and genetic factors in cancer, particularly colorectal cancer and fecal analysis.
- Cancer screenings in women (cervical and breast cancer) and men (prostate cancer), cancer risk groups, and the impact of age.
- The risk status of cancer in men and women, and the importance of guidelines and protocols.
- The significance of palliative care in cancer patients, combating cancer with comorbidities such as thyroid disease and diabetes.
- Addressing prostate, lung, and breast cancer, symptom management and diagnosis, and monitoring and controlling cancer survivors.

**Figure 3.**
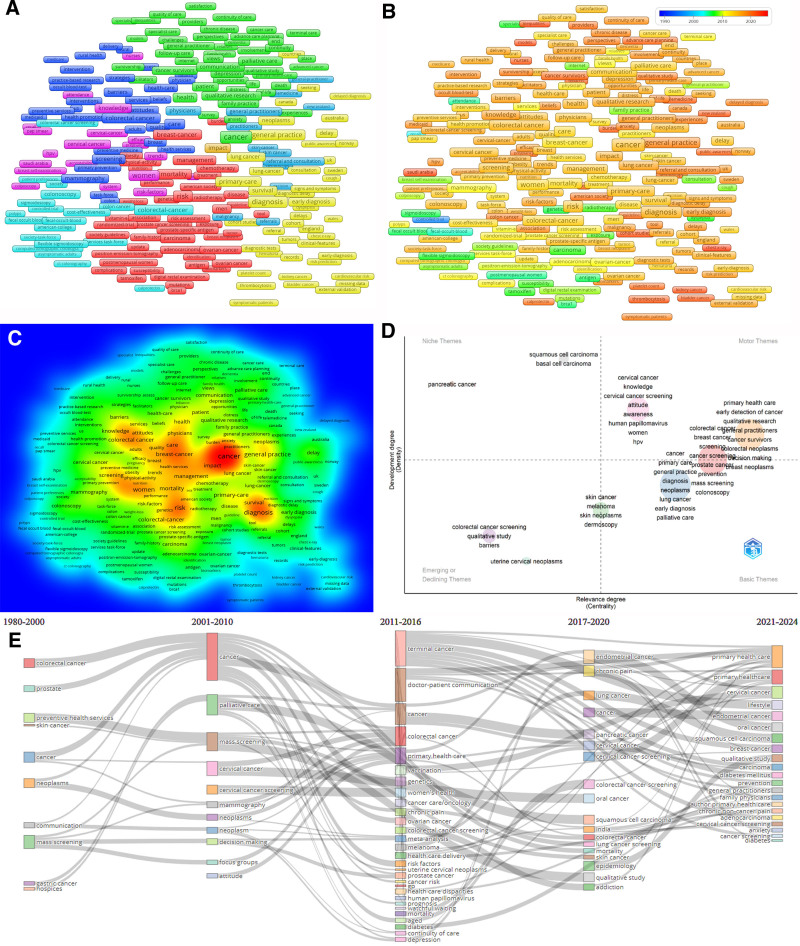
Keywords analyses (A. Co-occurence network analyses, B. Co-occurence overlay analyses, C. Co-occurence density analyses, D. Thematic map analyses, E. Thematic evolution).

Furthermore, Figure 3 illustrates the main keywords used in the articles through 5 different analytical charts presented in the subfigures. Figure 3A shows the class to which keywords are associated, Figure 3B shows the change in keywords over the years, and Figure 3C shows the density of keywords. The 4 distinct regions in Figure 3D correspond to different thematic significances: motor themes, indicating well-developed themes that are crucial for structuring a research topic; niche themes, signifying themes of limited importance; emerging or declining themes, suggesting minimally developed and marginal themes; and basic themes. Finally, Figure 3E shows these thematic relationships over the years showing how topics evolve and academic interest shifts. For example, between 1980 and 2010 primary healthcare studies on colorectal cancer mainly focused on its understanding, screening, and cancer care/treatment. Colorectal cancer was a high-trend topic between 2001 and 2016 with an emphasis on mass screening, the importance of different types of neoplasms, and medical decision-making around these topics. Beyond 2016, the attention on colorectal cancer shifted in other directions including its management in elderly patients, behavioral factors associated with colorectal cancer care (depression, anxiety), and comorbidities.

Figures highlight the most intensively studied cancer types in the PHC domain as colorectal, breast, cervical, prostate, lung, skin, ovarian, oral, endometrial, pancreatic, bladder, kidney, testicular, and gastric. Additionally, alongside these cancer types, prominent research topics in PHC include cancer screening, diagnosis, early detection, prevention, education, mass screening (also body index), genetic factors and family history, risk factors, assessment, symptoms/signs, preventive medicine, referral and consultation, continuity of patient care, chronic disease management for cancer patients, health services research for cancer patients, health care delivery, health care disparities, palliative care, and communication with patients in PHC.

## 4. Discussion

Our study represents one of the most comprehensive investigations exploring cancer-related research within the primary healthcare domain, for which a detailed literature review was lacking. This research provides an extensive evaluation for domain readers, cancer researchers, and PHC specialists. The results provided in the previous section demonstrate common and in-trend topics in this field. Based on these results (e.g., commonality and recent themes and topics) and the contents of the associated publications, the researchers specify 8 larger themes, summarize key findings in them, discuss the current status of the research around these larger themes, highlight in-trend issues and developments, and highlighted questions/challenges yet to be addressed.

### 4.1. Geographic variations and demographic discrepancies

Despite the geographical concentration of PHC research efforts in some countries such as the USA, England, Canada, and Australia, the universal challenge posed by cancer necessitates a concerted global response.^[13]^ The articles in the dataset were contributed by researchers from 75 different countries and have a high h-index with close to 10% international coauthorship.^[43]^ This shows a great interest in cancer studies in the PHC research field and some level of global collaboration. However, the rapid increase in new cancer cases represents a major potential crisis for public health and health systems around the world. This suggests that ensuring adequate funding for the treatment and care of all cancer patients will be a global challenge.^[44,45]^ Around one-third of all cancers are preventable primary prevention and early detection remain the most cost-effective strategies in cancer control.^[46,47]^ Therefore, more global collaboration around developing innovative ways of delivering primary care to patients at cancer risk and cancer survival is needed. This collaboration is believed to provide opportunities for achieving equality in the fight against cancer, especially for underdeveloped and developing countries. It is recommended that countries with strong PHC systems send researchers before and after doctoral training to countries with less activity in the field, in order to enhance scientific collaborations. Such initiatives will contribute to the faster development of primary health care services in these countries. These activities can be directly aligned with the WHO’s goals of Universal Health Coverage and Sustainable Development.

The key topics identified in our analyses indicate that cancer research within the primary healthcare domain has paid special attention to the issues related to minority health, access to healthcare services, and barriers to accessing health services due to poverty, and ethnography. The emphasis on these topics highlights the critical role of primary care in addressing socioeconomic and demographic disparities in healthcare. Cancer screening rates such as breast and cervical cancer are lower for Latin Americans compared to non-Hispanic white people.^[48,49]^ Factors such as being immigrants, lack of health insurance, and inadequate health literacy can significantly impact cancer outcomes. Thus, cancer mortality rates are higher among individuals with lower socioeconomic status compared to those with higher socioeconomic status, and this disparity is widening.^[50]^

Socioeconomic and demographic variables including gender, ethnicity, income, and education account for much of the observed disparities in cancer screening among different racial groups.^[51–53]^ For example, the delays in diagnosis and treatment have contributed to the high and steadily increasing cancer mortality rates in Latin America.^[54]^ Breast cancer is the most common cancer and the leading cause of cancer-related deaths among women in Latin America and the Caribbean. The high mortality-to-incidence ratio in these regions is primarily attributed to the high rates of late-stage diagnosis, which are closely linked to inadequate access to healthcare services.^[54]^ African Americans have very high disease and mortality rates in the USA.^[55]^ In developing countries, the incidence of stomach cancer is significantly higher, ranking second only to lung cancer in terms of mortality rates.^[56,57]^

Today, due to non-ending global conflicts immigrants are a reality in all countries, therefore, the effects of these demographic and socioeconomic discrepancies will be experienced more intensively across the world in the future.^[58,59]^ Even in multicultural societies with large immigrant populations, such as Canada, the UK, and the USA, minorities and immigrants need to have more effective access to primary healthcare services. Developing a strong and fair primary care system could be a reliable and efficient way of addressing such discrepancies given its cost-effectiveness and existing evidence pointing out more equitable specialized care outcomes in countries with well-developed primary care.^[46,60,61]^

### 4.2. Physicians’ role in cancer prevention and the use of decision support tools in primary health care

Cancer diagnosis and screening, the monitoring and care of cancer patients are increasingly becoming an integral part of family physicians’ practices. During this process, family physicians should strive to enhance their ability to provide emotional support to patients for more effective communication.^[62,63]^ In our study, communication in patient care emerged as a significant theme. Family physicians improving communication can enhance patient relationships, boosting treatment adherence and health outcomes. Lasser et al^[64]^ identified that a lack of trust in doctors and the absence of physician recommendations are significant barriers to cancer screening. From the screening process to diagnosis, treatment, and follow-up, it is the responsibility of primary care physicians to alleviate patients’ concerns and provide high-quality care through effective communication.^[65,66]^ The motivation of doctors working in the PHC domain is also critically important in this context. Although additional payments are the first motivational tool that comes to mind, Kiran et al^[67]^ advised policymakers to consider alternative strategies beyond financial incentives, noting that fee policies did not improve cancer screening rates.

Most cancer patients present with symptoms to PHC services.^[11,68]^ However, these cancer symptoms often pose diagnostic challenges for family physicians due to their commonality and lack of specificity.^[45,69]^ There are many types of cancer, each presenting different symptoms. Nine out of 10 cancer patients present to a healthcare facility with symptoms, and the majority of these patients initially seek care within PHC services.^[11]^ However, challenges such as physician time constraints, concerns about wasting time, long patient waiting times, and high patient volumes can complicate efforts in combating cancer.^[70]^

Addressing these challenges requires better cancer diagnoses, optimizing workflows, and leveraging technology to enhance efficiency. Our findings highlighted decision support tools, as a prominent topic within cancer research in the PHC. These tools can analyze patient data (e.g., from electronic health records), including symptoms and medical history, to provide evidence-based recommendations and alert clinicians to potential cancer cases faster and more accurately. Advanced scheduling algorithms can help manage patient appointments more effectively, reducing waiting times and ensuring that physicians can allocate adequate time for each consultation. Thus, the integration of decision support tools in PHC can significantly improve overall cancer care outcomes. It is thought that decision support tools, supported by global health organizations (e.g., WHO), which provide global services for free to all countries, could play a significant role in addressing inequalities based on sociodemographics, payment types, or living in rural areas in healthcare. Furthermore, rapidly developing artificial intelligence tools are expected to facilitate such initiatives. Birinci basamak hekimlerinin de bu sayede işi oldukça kolaylaşabilir.

Braithwaite et al^[71]^ recommended decision support tools as an effective method for primary care physicians to manage cancer in family histories. Dikomitis et al^[72]^ stated that decision support tools can assist in recognizing potential cancer symptoms and can be utilized by general practitioners. Additionally, the literature includes decision support tools for various cancer types that consider factors such as age, smoking status, alcohol consumption, chronic pancreatitis, type 2 diabetes, loss of appetite, weight loss, and abdominal pain.^[73–75]^ The critical value of these systems lies in their ability to seamlessly integrate into the healthcare system, facilitating both patient and physician interactions, and becoming a natural component of healthcare delivery.

### 4.3. Childhood cancers and primary health care services

Childhood cancers pose unique challenges within PHC and require a specialized approach, which we found to be intensively studied. Early detection and timely intervention are crucial for improving survival rates and outcomes in pediatric cancer patients. Approximately 400,000 children are diagnosed with cancer each year.^[76]^ The most commonly diagnosed cancers in children and adolescents are leukemia (children: 28%, adolescents: 13%), brain tumors including benign and borderline malignant tumors (children: 26%, adolescents: 21%), and lymphomas (children: 12%, adolescents: 19%). The childhood cancer burden, which has been largely underestimated in the past, can be effectively reduced to achieve immense health and economic benefits and prevent millions of unnecessary deaths.^[77]^

Childhood cancers are challenging because they often present nonspecific symptoms and there are a lot of controversies about the right treatment and dosage due to higher variability in weight and body-mass-index. This leads to delays in diagnosis and the initiation of appropriate treatment.^[78]^ Thus, along with pediatricians, the vigilance of primary care is particularly against childhood cancers. A cautious and skeptical approach by primary care physicians can lead to earlier diagnosis and treatment, especially in children presenting with persistent symptoms and signs. Erdmann et al^[79]^ and Atun et al^[77]^ stated that that with advances in treatment, survival rates in childhood cancers have increased and this increase is greater in cases diagnosed early. This growing population requires special attention and care from primary healthcare professionals as childhood cancer survivors often face long-term health issues related to their cancer treatment to be addressed within primary and pediatric care.

### 4.4. Commonly analyzed cancers in primary health care research

The relevant research in the primary healthcare domain especially focuses on breast, skin, cervical, lung cancer, and colorectal cancers. Each of these types is associated with different challenges. Mammography is a vital tool for early breast cancer detection. However, screenings do not always yield accurate results, and false positives can have long-term psychosocial impacts on patients. Brodersen and Siersma^[80]^ emphasized that women are significantly affected by false positive findings. Therefore, it is recommended that health professionals in primary care incorporate processes to manage and mitigate the effects of false positives within their care services.

Cervical cancer is the fourth most common cancer in women worldwide ranking after breast cancer (2.1 million cases), colorectal cancer (0.8 million), and lung cancer (0.7 million). Women most susceptible to cervical cancer are those who miss regular screenings or have never been screened.^[81]^ While pap smear test is the most commonly used tool for combating this cancer, promi sing new screening methods have been developed, e.g., the HPV DNA test. Screening has significantly reduced the incidence and mortality rates of cervical cancer in women.^[82]^ Factors contributing to low screening rates include low income, low education, minority status, lack of cancer knowledge, attitudes and beliefs, shame, language barriers, specific cultural beliefs, and sexual trauma.^[83–85]^ To overcome these barriers, it is crucial to enhance the quality of screening services, provide patients with additional information and education about the tests, and establish effective communication with patients within the PHC setting.

The incidence of skin cancer is increasing at epidemic rates. Basal cell carcinoma remains the most common skin neoplasm. However, skin lesions are often ignored as most are benign, however, the malignant ones are deadly. Sun exposure continues to be the most significant risk factor for all skin neoplasms. Therefore, primary care practitioners must have a higher awareness of skin cancer risk, and educate patients on basic sun safety measures, especially for fair-skinned populations with higher vulnerability to skin cancer.^[86,87]^

The early detection of lung cancer is challenging due to the nonspecific nature of most symptoms, which often resemble common benign respiratory conditions.^[88]^ Mitchell et al^[89]^ highlighted that chest symptoms and smoking habits are significant indicators, with lung cancer patients frequently presenting with chest infections and coughs. Routine implementation of an effective screening test for early-stage lung cancer is vital,^[90]^ especially considering that 79% of lung cancers are diagnosed at regional or distant stages.^[91]^ Therefore, identifying high-risk patients requiring further investigation is a critical responsibility for primary care health professionals.

Colorectal cancer is the third most common cancer worldwide and largely preventable through changes in modifiable risk factors, alongside the detection and removal of precancerous lesions.^[92]^ The incidence and mortality of colorectal cancer are low until middle age, after which they increase rapidly.^[93]^ Despite the availability of effective screening tests, a significant portion of Americans still do not undergo colorectal cancer screening. High-risk groups for non-screening include racial and ethnic minorities, uninsured patients, foreign-born individuals, and those with low socioeconomic status.^[94]^ Greater attention should be given to these social groups in countries with large minority and immigrant populations, such as the UK, Canada, Australia, and the USA. Gandhi et al^[95]^ emphasized the need to continue supporting cancer screening programs among underserved minorities.

### 4.5. Early diagnosis and prevention of cancer in primary health care

Cancer prevention and early diagnosis were another 2 heavily debated topics. Cancer is a disease with many types, each presenting with unique symptoms, though some symptoms may overlap across different cancer types. For instance, the most common symptom of endometrial cancer is postmenopausal bleeding, making it the most prevalent gynecologic malignancy.^[96]^ While ultrasound is the initial investigative choice for kidney cancer, cystoscopy is preferred for bladder cancer. Both procedures can be conducted in most urology clinics, allowing general practitioners to test for potential kidney cancer without a referral, at least in theory.^[97]^ Most prostate cancers are diagnosed based on lower urinary tract symptoms.^[98,99]^ Alarm symptoms such as hematemesis, dysphagia, weight loss, loss of appetite, or abdominal pain may signal existing gastro-esophageal cancer.^[100,101]^ Common symptoms of esophageal cancer include dysphagia, odynophagia, and progressive weight loss.^[5]^ For colorectal and lung cancers, weight loss can be a symptom of both early and late-stage cancers.^[102]^

As you can see, there are different symptoms for each type of cancer and the primary care doctor’s job is very difficult at this point. However, decision support tools integrated into the systems used, well-constructed guidelines, continuous information, and awareness training can facilitate this process. Shapley et al^[103]^ identified 8 symptoms and non-diagnostic test results as strong indicators of cancer for specific age and gender groups in their literature review. Thrombocytosis is an early indicator of certain cancers in primary care settings, and thrombocytosis is a predictor of cancer across all areas, including breast cancer, colorectal cancer, lung cancer, ovarian cancer, bladder cancer, kidney cancer, pancreatic cancer, esophagogastric cancer, and uterine cancer.^[96,104]^ Age,^[105]^ physical activities and exercises,^[16,106,107]^ sedentary lifestyle,^[106,108]^ nutrition^[109]^ are also an important factor in cancer screening by primary care physicians. It may be recommended to be more careful and attentive to the health care of individuals in need of special care, especially the elderly and disabled individuals.^[108,110,111]^ In line with WHO’s Universal Health Coverage goals and Sustainable Development Goals, AI-based solutions in primary healthcare have great potential. By increasing access to healthcare, AI can reduce health inequalities and improve the efficiency of healthcare systems. AI-based health screenings and early detection systems can be integrated into primary healthcare, especially in underdeveloped and developing regions. This would allow common health problems such as cancer, diabetes and cardiovascular diseases to be detected in the early stages, enabling more effective interventions. Furthermore, digital health platforms and telemedicine applications can facilitate access to healthcare for individuals in remote areas. AI-supported decision support systems can accelerate the diagnosis and treatment decisions of primary healthcare professionals, ensuring efficient use of resources. These practices increase accessibility to health services within the framework of Universal Health Coverage targets and contribute to the Sustainable Development Goals, particularly on health equity and access, quality health services and efficient use of resources. In conclusion, we have enough evidence for data-driven decision-support tools and AI-Based tools for improving cancer detection accuracy and efficiency, leading to better patient outcomes and more effective cancer management in PHC.

### 4.6. Cancer cases in rural areas and primary health care

Patients living in rural and remote areas often need to travel to larger centers for cancer care and rely heavily on PHC providers to maintain continuity of care.^[112]^ Cancer incidence in rural areas presents unique challenges and necessitates targeted strategies within PHC. Rural populations often face barriers such as limited access to healthcare facilities, and lower socioeconomic status. For patients, the essential components of cancer care are similar whether they reside in rural or urban areas. However, rural patients often face greater barriers to accessing specialist care.^[113,114]^ These differences can lead to significant delays, resulting in more advanced stages of cancer at diagnosis for rural patients.^[115–117]^ Family physicians are in an ideal position to assess familial cancer risk, refer to cancer clinics, provide counseling, and discuss and plan appropriate preventive and health promotion strategies for patients at risk of hereditary cancer.^[9]^ However, considering the challenges faced by rural patients and physicians in accessing urban healthcare services, implementing appropriate decision support tools and educational resources via mobile or health information systems can mitigate some of the disadvantages associated with accessing health services in rural areas. AI technologies can provide great benefits such as early screening, remote monitoring, personalized treatment and decision support for health professionals. For instance, AI-powered imaging systems (especially deep learning based systems for very rare diseases) and remote diagnostic tools can help healthcare professionals in rural areas detect serious conditions such as cancer or cardiovascular diseases in the early stages, even in non-specialized areas. Furthermore, AI can facilitate access to healthcare for patients in remote areas by enabling them to see specialized doctors through telemedicine applications. However, successful implementation of these technologies requires overcoming barriers such as infrastructure, education, access to technology, national or regional health policy development and cultural adaptation. In this context, while AI has great potential to improve cancer care in rural areas, effective implementation requires the right strategies and investments.

### 4.7. Family history, genetic factors, and their impact on primary health care

Family history is a relatively simple and accurate method for classifying cancer risk for many significant cancers.^[118]^ Professional organizations recommend using family history information for cancer risk assessment.^[16]^ Family health history is a strong predictor of disease risk^[119]^ but is significantly underutilized in primary care.^[120]^ In our research, family history and genetic factors emerged as frequently discussed and researched topics in primary care. A detailed family history can help assess and manage risk for various cancers and can be used as a reference for cancer screening.^[121]^ Advances in cancer genetics and increased public awareness of the importance of family cancer history indicate that primary care physicians will increasingly be responsible for assessing family history information.^[122]^ However, current evidence shows that family histories typically taken in primary care are inadequately evaluated.^[120,121]^ Additionally, challenges have been encountered in the collection and utilization of genetic factors.^[123]^ To overcome these challenges, decision support systems and AI-based systems that calculate genetic risk factors through a centralized system could have a significant impact on cancer management, particularly in PHC. Integrating advanced analytics and decision support systems into primary care practices can enhance the utilization of family history for cancer risk assessment.

### 4.8. Continued care for cancer survivors within primary health care

The literature indicates that cancer survivors place a significantly higher burden on primary care compared to other patients. Post-cancer health issues encompass a broad spectrum, including infections, chronic diseases, minor illnesses, sleep disorders, and psychosocial problems such as depression and anxiety.^[124]^ In our study has shown that cancer survivorship care and its integration into primary healthcare have emerged as prominent issue in our work. Cancer survivors face significant risks of late mortality, morbidity, and adverse health outcomes due to previous cancer treatments.^[78,125]^ For example, it is estimated that by 2030, there will be 22.1 million cancer survivors in the United States.^[126]^ Psychological distress in cancer patients, including depression and anxiety, ranges from 0% to 44%.^[127]^ Additionally, cancer survivors require psychosocial rehabilitation. Mikkelsen et al^[128]^ emphasized that if this task is assigned to general practitioners, they need to be proactive in assessing psychosocial aspects. Another prominent and debated topic in the literature is the follow-up of cancer patients by primary care physicians. In some countries, including Norway, follow-up care for cancers with a low risk of recurrence, such as breast and colorectal cancer, is already conducted in primary care settings.^[128]^ Therefore, in light of all these findings, it is deemed necessary for primary healthcare physicians to specialize further in the field of cancer. National solutions can be implemented at this point. National or regional artificial intelligence-based telemedicine service solutions can be used for health services that cancer patients who have had cancer or cancer patients need to receive from primary care. In this way, the training needs of hemics can be met. Patients can be prevented from being victimized due to incomplete or unspecialized services.

## 5. Strength and limitation

This study represents the first bibliometric analysis conducted on cancer in the PHC research field as well as the first bibliometric analysis conducted using machine learning techniques. However, our study had some limitations. First, all data were obtained from the WoS Core Collection. Although WoS indexes the most important journals in the field, there may be journals that are not. Some journals indexed in local journals and Scopus have been overlooked. Second, our research only analyzed articles and review article types. The primary reason is that these document types are the most suitable for providing the essence of the field, and they are the richest bibliometric data types in terms of metrics such as abstracts, titles, keywords, and references. Thirdly, although less-developed and developing countries demonstrate scientific productivity in leading journals within the field, their scientific output lags far behind countries such as Canada, the United Kingdom, and the United States. However, these studies do not reveal the specific context and prominent topics of these countries. This gap, however, could be addressed in future research. Future studies could explore cancer research within the field of primary healthcare services through local journals or other bibliometric databases such as Scopus. A particular focus could be placed on the primary healthcare research conducted in less-developed and developing countries. Additionally, analyses of scientific productivity and trend topics could be conducted regionally and at the country level. This approach could provide valuable policy recommendations for primary healthcare services in the relevant geographical areas.

## 6. Conclusion

PHC plays a crucial and pivotal role in the fight against cancer within the healthcare system today. This article represented one of the most comprehensive studies on cancer research in PHC research area, evaluating all aspects of cancer within this context. The literature indicates that cancer survivors are more vulnerable and require more sensitive health services. Family physicians, frequently in front-line contact with the public, play a vital role in the primary prevention of cancer. Detailed analysis shows that family physicians are critical in the care of patients with active cancer diagnoses, the ongoing life management of cancer survivors, and the early detection and referral of cancer cases. Additionally, the role of family physicians in the successful management of cancer within the healthcare system is substantial. It is crucial that they are aware of this role, possess up-to-date knowledge based on the latest developments in the literature, and guide patients toward early diagnosis and treatment. Providing appropriate health education in primary care, preparing and distributing suitable educational materials, and raising patient awareness can positively influence attitudes toward cancer screenings.

The literature provides substantial evidence regarding the effective use of data-driven decision support tools in the fight against cancer. In addition, recent advancements in big data, the Internet of Things (IoT), increased internet speeds, and technologies such as artificial intelligence (AI) and blockchain offer great potential for national, regional, and global health solutions. In particular, progress in deep learning and image processing facilitates the diagnosis of cancer and other diseases where image processing is critical. Additionally, advancements in server technologies allow for easier and faster storage of larger volumes of data, internet access is now readily available in nearly every country, enabling access to information and services, and blockchain technology ensures more secure data storage. These technological advancements and capabilities collectively facilitate the provision of macro solutions for global health challenges, such as cancer, and require their rapid deployment.

Moreover, such solutions are believed to enhance the ability of underdeveloped and developing countries to effectively tackle global health issues, contributing to the reduction of inequalities in access to healthcare services in these regions. National, regional, or global health authorities (e.g., WHO) could design low-cost and accessible AI solutions (e.g., image processing services, telemedicine services, virtual assistants for assistance and support, health data analysis services, screening tools, decision support tools for physicians), allowing broader access to healthcare services in rural and underdeveloped areas. These types of applications could play a crucial role in addressing health inequalities in rural areas or developing countries. Furthermore, to expedite the development of primary health care (PHC) research in developing countries, academic resources from advanced leading countries can be leveraged. National health policies can be implemented to foster the development of PHC, facilitating collaboration between researchers from leading institutions and those from underdeveloped countries. This would accelerate the global collaboration in the healthcare field.

## Acknowledgments

M. Damar was supported by the Scientific and Technological Research Council of Türkiye (TUBITAK) under the TUBITAK 2219 International Postdoctoral Research Fellowship program. He would like to thank the Upstream Lab, MAP, Li Ka Shing Knowledge Institute at the University of Toronto for its excellent hospitality. The authors have no conflicts of interest to disclose.

## Author contributions

**Conceptualization:** Hale Turhan Damar, Şeyda Özbiçakci, Gökben Yasli, Fatih Safa Erenay, Güzin Özdağoğlu, Andrew David Pinto.

**Data curation:** Muhammet Damar, Hale Turhan Damar, Şeyda Özbiçakci, Andrew David Pinto.

**Formal analysis:** Muhammet Damar, Hale Turhan Damar, Şeyda Özbiçakci, Gökben Yasli, Andrew David Pinto.

**Funding acquisition:** Muhammet Damar, Hale Turhan Damar.

**Investigation:** Muhammet Damar, Hale Turhan Damar, Şeyda Özbiçakci, Gökben Yasli.

**Methodology:** Muhammet Damar, Hale Turhan Damar, Fatih Safa Erenay, Güzin Özdağoğlu.

**Project administration:** Muhammet Damar.

**Resources:** Muhammet Damar, Şeyda Özbiçakci, Gökben Yasli, Güzin Özdağoğlu.

**Software:** Muhammet Damar.

**Supervision:** Muhammet Damar, Şeyda Özbiçakci, Gökben Yasli, Fatih Safa Erenay, Andrew David Pinto.

**Validation:** Muhammet Damar, Hale Turhan Damar, Gökben Yasli, Fatih Safa Erenay, Andrew David Pinto.

**Visualization:** Muhammet Damar, Hale Turhan Damar, Şeyda Özbiçakci, Fatih Safa Erenay, Güzin Özdağoğlu, Andrew David Pinto.

**Writing – original draft:** Muhammet Damar, Hale Turhan Damar, Şeyda Özbiçakci, Gökben Yasli, Fatih Safa Erenay, Güzin Özdağoğlu, Andrew David Pinto.

**Writing – review & editing:** Muhammet Damar, Hale Turhan Damar, Şeyda Özbiçakci, Gökben Yasli, Fatih Safa Erenay, Güzin Özdağoğlu, Andrew David Pinto.

## Supplementary Material

SUPPLEMENTARY MATERIAL
