# Supplementary material for: Mapping intellectual structure and research hotspots of cancer studies in primary health care: A machine-learning-based analysis
Source: Medicine (Baltimore). 2025 Mar 21;104(12):e41749. doi: 10.1097/MD.0000000000041749 (PMC11936571; doi:10.1097/MD.0000000000041749)
Supplement: SUPPLEMENTARY MATERIAL [file medi-104-e41749-s001.docx]

**Appendix 1.** The Distribution of the Number of Articles by Authors in PHC Research Field on Cancer

| **Rank** | **Authors** | **Affiliations** | **TC*** | **HI** | **ACPA** | **N** |
| --- | --- | --- | --- | --- | --- | --- |
| 1 | Hamilton, Willie | University of Exeter Medical School | 1,697 | 24 | 26.52 | 64 |
| 2 | Vedsted, Peter | Aarhus University | 568 | 11 | 15.78 | 36 |
| 3 | Emery, Jon D. | University of Melbourne | 710 | 12 | 21.52 | 33 |
| 5 | Walter, Fiona | University of Cambridge | 739 | 13 | 23.09 | 32 |
| 6 | Neal, Richard | University of Exeter | 761 | 14 | 31.71 | 24 |
| 7 | Weller, David | University of Edinburgh | 521 | 12 | 22.65 | 23 |
| 8 | Rose, Peter | University of Nottingham | 482 | 13 | 24.10 | 20 |
| 9 | Søndergaard, Jens js | University of Southern Denmark | 358 | 10 | 19.89 | 18 |
| 10 | Murchie, Peter | University of Aberdeen | 285 | 12 | 16.76 | 17 |
| 11 | Nicholson, Brian D. | University of Oxford | 243 | 9 | 15.19 | 16 |
| 12 | Macleod, Una | University of Hull | 487 | 13 | 32.47 | 15 |
| 13 | Olesen, F. | Aarhus University | 515 | 11 | 34.33 | 15 |
| 14 | Watson, Eila | Oxford Brookes University | 420 | 12 | 30.00 | 14 |
| 15 | Lofters, Aisha | University of Toronto | 329 | 10 | 23.50 | 14 |
| 16 | Jensen, Henry | Aarhus University | 91 | 6 | 7.00 | 13 |
| 17 | Levy, Barcey T. | University of Iowa | 215 | 8 | 16.54 | 13 |
| 18 | Abel, Gary | University of Exeter | 234 | 7 | 18.00 | 13 |
| 19 | Lyratzopoulos, Georgios | University College London | 242 | 8 | 18.62 | 13 |
| 20 | Rubin, G. | Newcastle University | 378 | 9 | 31.50 | 12 |
| 21 | Jarbøl, Dorte E | University of Southern Denmark | 117 | 6 | 9.75 | 12 |
| 22 | Grunfeld, Eva | University of Toronto | 480 | 9 | 40.00 | 12 |
| 23 | Daly, J. M. | University of Iowa | 171 | 7 | 15.55 | 11 |
| 24 | Carroll, June C | University of Toronto | 256 | 9 | 25.60 | 10 |
| 25 | Ferrante, Jeanne | Rutgers University | 443 | 9 | 44.30 | 10 |
| 26 | Hippisley-Cox, Julia | University of Oxford | 407 | 8 | 40.70 | 10 |
| **Co-authorship Authors Network Analysis** | | | | | | |
| 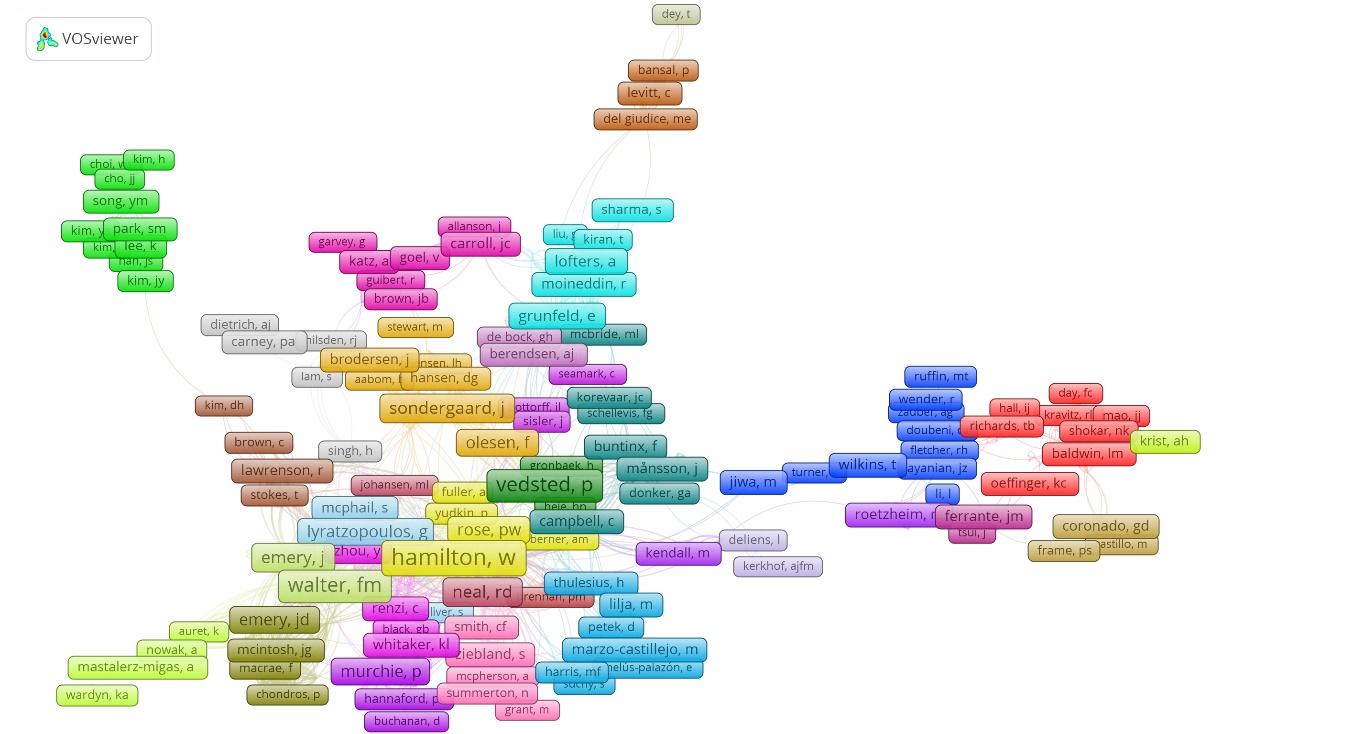 | | | | | | |

**ACPA: Average Citation per Articles, N= Document Count, HI: H-index, TC: Times Cited*

***Prepared The List According to Authors’ Full Name According to Web of Science Researcher Profile*
