# Supplementary material for: Mapping intellectual structure and research hotspots of cancer studies in primary health care: A machine-learning-based analysis
Source: Medicine (Baltimore). 2025 Mar 21;104(12):e41749. doi: 10.1097/MD.0000000000041749 (PMC11936571; doi:10.1097/MD.0000000000041749)
Supplement: SUPPLEMENTARY MATERIAL [file medi-104-e41749-s002.docx]

**Appendix 2.** The Distribution of the Number of Articles by Institutions in PHC Research Field on Cancer

| **Rank** | **Affiliations** | **Country** | **TC** | **HI** | **ACPA** | **N** | **%** |
| --- | --- | --- | --- | --- | --- | --- | --- |
| 1 | University of Exeter | England | 1,571 | 24 | 19.4 | 81 | 3.97 |
| 2 | University of London | England | 1,466 | 21 | 18.56 | 79 | 3.87 |
| 3 | University of Toronto | Canada | 1,514 | 21 | 20.19 | 75 | 3.67 |
| 4 | University of California System | USA | 2,184 | 21 | 37.02 | 59 | 2.89 |
| 5 | University of Oxford | England | 1,482 | 23 | 27.44 | 54 | 2.64 |
| 6 | University of Cambridge | England | 1,214 | 18 | 23.8 | 51 | 2.50 |
| 7 | Aarhus University | Denmark | 798 | 15 | 16.63 | 48 | 2.35 |
| 8 | University of Melbourne | Australia | 349 | 10 | 8.73 | 40 | 1.96 |
| 9 | University of Sydney | Australia | 336 | 11 | 9.08 | 37 | 1.81 |
| 10 | University of Texas System | USA | 1,573 | 22 | 42.51 | 37 | 1.81 |
| 11 | University of Edinburgh | Scotland | 906 | 16 | 25.17 | 36 | 1.76 |
| 12 | University College London | England | 531 | 13 | 15.17 | 35 | 1.71 |
| 13 | University of Southern Denmark | Denmark | 425 | 11 | 12.50 | 34 | 1.66 |
| 14 | State University System of Florida | USA | 551 | 13 | 17.22 | 32 | 1.56 |
| 15 | Queen Mary University London | England | 236 | 8 | 7.87 | 30 | 1.47 |
| **Co-authorship Institutions Overlay Analysis** | | | | | | | |
| 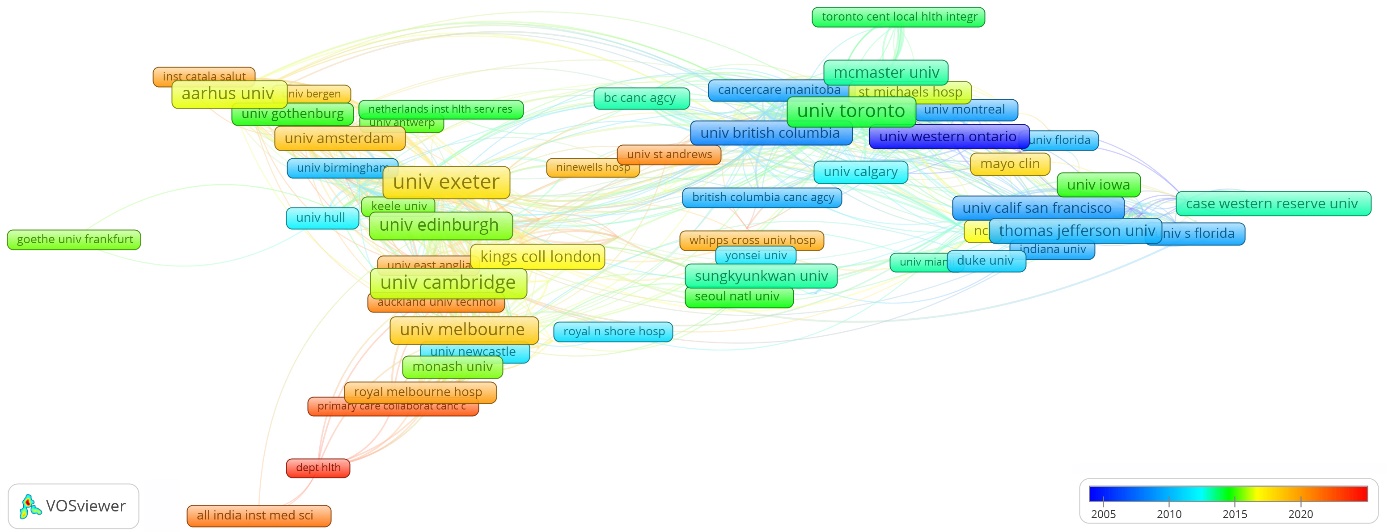 | | | | | | | |
| **Co-authorship Institutions Network Analysis** | | | | | | | |
| 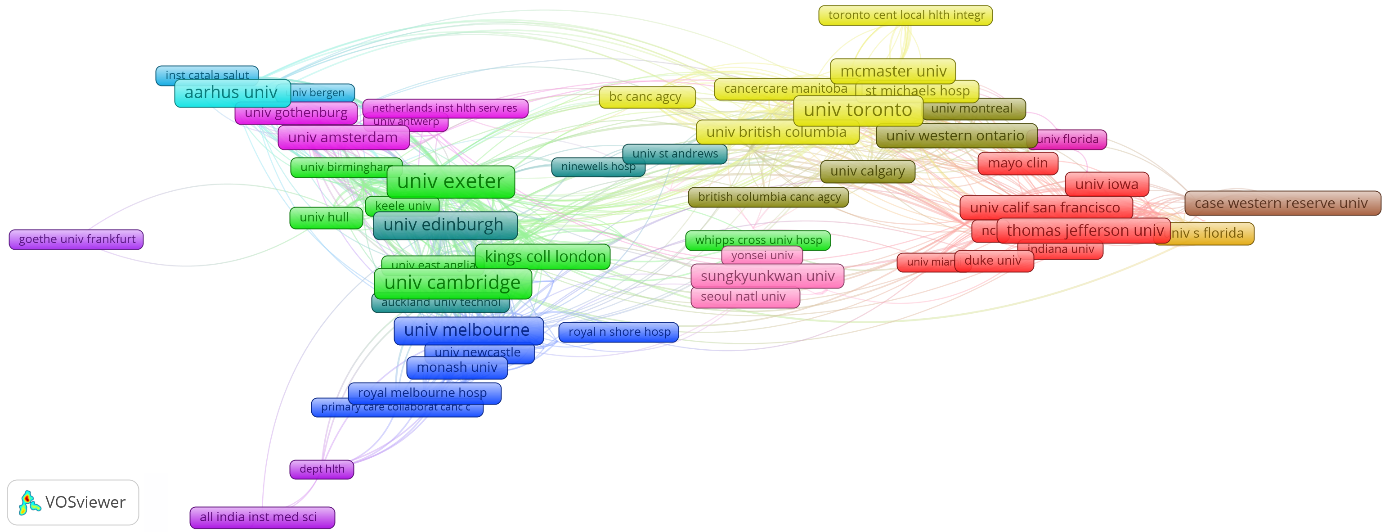 | | | | | | | |

**ACPA: Average Citation per Articles, N: Document Count, HI: H-index, TC: Times Cited*
