# Supplementary material for: Mapping intellectual structure and research hotspots of cancer studies in primary health care: A machine-learning-based analysis
Source: Medicine (Baltimore). 2025 Mar 21;104(12):e41749. doi: 10.1097/MD.0000000000041749 (PMC11936571; doi:10.1097/MD.0000000000041749)
Supplement: SUPPLEMENTARY MATERIAL [file medi-104-e41749-s003.docx]

**Appendix 3.** The Distribution of the Number of Articles by Countries in PHC Research Field on Cancer

| **Rank** | **Countries** | **HI** | **ACPA** | **N** | **%** | **Rank** | **Countries** | **HI** | **ACPA** | **N** | **%** |
| --- | --- | --- | --- | --- | --- | --- | --- | --- | --- | --- | --- |
| 1 | USA | 59 | 21.47 | 716 | 35.09 | 16 | Saudi Arabia | 5 | 4.04 | 24 | 1.17 |
| 2 | England | 45 | 23.95 | 318 | 15.58 | 17 | Norway | 10 | 11.87 | 23 | 1.12 |
| 3 | Canada | 34 | 16.56 | 224 | 10.98 | 18 | Belgium | 12 | 19.86 | 21 | 1.02 |
| 4 | Australia | 21 | 10.18 | 166 | 8.13 | 19 | France | 8 | 10.75 | 20 | 0.98 |
| 5 | Poland | 4 | 0.69 | 88 | 4.31 | 20 | Germany | 6 | 8.53 | 19 | 0.93 |
| 6 | Denmark | 20 | 17.8 | 84 | 4.11 | 21 | South Africa | 6 | 5.07 | 15 | 0.73 |
| 7 | Netherlands | 19 | 13.88 | 82 | 4.02 | 22 | Ireland | 6 | 11.64 | 14 | 0.68 |
| 8 | Scotland | 21 | 22.01 | 69 | 3.38 | 23 | Italy | 5 | 14.58 | 12 | 0.58 |
| 9 | India | 7 | 3.03 | 65 | 3.18 | 24 | China | 5 | 8.78 | 9 | 0.44 |
| 10 | South Korea | 10 | 8.45 | 62 | 3.03 | 25 | Switzerland | 3 | 2.22 | 9 | 0.44 |
| 11 | Sweden | 14 | 12.14 | 43 | 2.10 | 26 | Israel | 4 | 8.63 | 8 | 0.39 |
| 12 | Spain | 9 | 7.9 | 40 | 1.96 | 27 | Japan | 3 | 1.75 | 8 | 0.39 |
| 13 | New Zealand | 11 | 10.20 | 30 | 1.47 | 28 | Finland | 2 | 17.50 | 6 | 0.29 |
| 14 | Wales | 15 | 22.85 | 27 | 1.32 | 29 | Austria | 3 | 3.80 | 5 | 0.24 |
| 15 | Iran | 4 | 2.65 | 26 | 1.27 | 30 | Nigeria | 4 | 5.00 | 5 | 0.24 |
| **Co-authorship Countries Network Analysis** | | | | | | | | | | | |
| 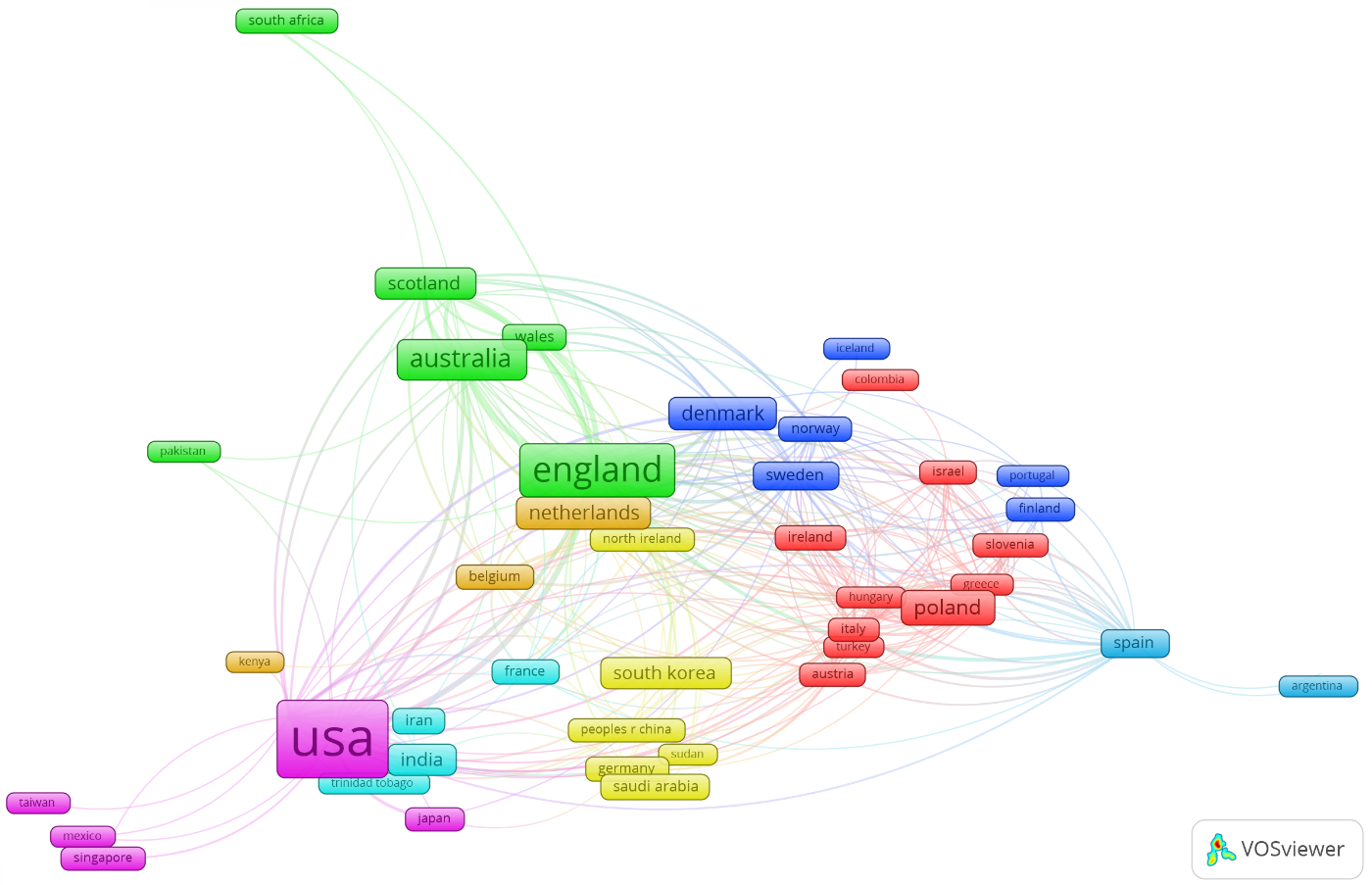 | | | | | | | | | | | |

**ACPA: Average Citation per Articles, N: Article Count, HI: H-Index*
